# Isolation, characterization, and genomic analysis of novel bacteriophage AEV23 against multidrug-resistant *Klebsiella pneumoniae* isolated from Cystic Fibrosis patients

**DOI:** 10.1186/s12866-025-04243-6

**Published:** 2026-02-18

**Authors:** Atefe Jafari, Ameneh Elikaei, Erfan Gowdini, Ahya Abdi Ali, Sepideh Meidaninikjeh

**Affiliations:** 1 Department of Microbiology, Faculty of Biological Sciences, Alzahra University, Tehran, Iran; 2 Department of Molecular Medicine, National Institute of Genetic Engineering and Biotechnology, Tehran, Iran

**Keywords:** Phage, *K. pneumoniae*, CF, MDR, ESBL, Biofilm, Whole genome sequencing

## Abstract

**Background:**

*Klebsiella pneumoniae* is a well-known opportunistic pathogen in humans and can cause chronic obstructive pulmonary disease (COPD) in Cystic Fibrosis (CF) patients. This pathogen has developed resistance to *β-lactam* antibiotics due to the expression of extended-spectrum beta-lactamase (ESBL) genes, leading to current treatment challenges in these patients. Bacteriophages are alternative and effective treatment options against multidrug-resistant (MDR) pathogens. In this study, a lytic bacteriophage was isolated from untreated sewage, tested against multidrug-resistant *K. pneumoniae* strains, and evaluated for its therapeutic potential *in vitro.*

**Methods:**

This study presents the microbiological, physicochemical, and genomic characterization of a virulent bacteriophage. The phage was studied by electron microscopy, host range analysis, multiplicity of infection (MOI) determination, adsorption rate measurement, burst size calculation, latent period assessment, stability testing to temperature, chloroform, pH and salt stress, and biofilm removal ability evaluation demonstrated by SEM; the bacteriophage genome was studied by complete genome sequencing.

**Results:**

The phage exhibited a broad and highly specific host range for *K. pneumoniae* strains. Its stability under stress conditions, including changes in temperature, pH, salt concentration, and exposure to chloroform, was 64.63%, 52.79%, 68.36%, and 98.92%, respectively. The one-step growth curve results demonstrated that the bacteriophage had a latent period of 30 min and a burst size of approximately 98 plaque-forming units per infected cell (PFU/cell). Adsorption assays revealed that 92% of isolated phages adsorbed to bacterial cells within 5 min. Additionally, the bacteriophage showed inhibitory activity against bacterial growth at an MOI 1. The biofilm removal assay demonstrated that the phage eliminated over 93% of the cellular biomass, as confirmed by scanning electron microscopy (SEM). Whole-genome analysis showed that it belongs to the *Loughboroughvirus* genus. The phage has a linear, double-stranded DNA genome with a length of 55,637 bp and a GC content of 45.9%. The genome encodes 76 open reading frames (ORFs), including structural proteins, DNA metabolism enzymes, lysis modules, and packaging proteins. No tRNAs, lysogenic genes, or virulence factors were detected in the genome.

**Conclusions:**

Characterization of phage VB_KpM-AEV23 demonstrated its high host specificity against *K. pneumoniae*, making it a suitable candidate for phage therapy applications.

## Introduction

Mutations in the Cystic Fibrosis Transmembrane Regulator (CFTR) gene cause the genetic disorder Cystic Fibrosis (CF). This gene regulates chloride and sodium ion movement across epithelial membranes [1]. It significantly affects the pancreas, lungs, and other organs, as well as the liver, intestine, upper airways, and reproductive organs’ functions [2]. An estimated 70,000–100,000 individuals worldwide have CF, while more recent data suggest approximately 162,428 people are currently living with CF globally. This condition significantly impacts global health and adversely affects patients’ quality of life. However, advances in medical treatments have reduced CF-associated mortality rates. In high-income countries, the median survival age of individuals with Cystic Fibrosis has increased to 40–50 years, with some patients surviving into their 60 s or beyond. However, life expectancy varies substantially depending on disease severity, healthcare access, and treatment adherence [3–5]. The respiratory systems of CF patients are colonized by numerous bacterial species from early childhood. Members of the *Enterobacteriaceae* family can transiently colonize the airways of CF patients. The isolation of multi-drug resistant (MDR) *K. pneumoniae* from the respiratory tract of CF patients, along with the transient or non-transient establishment of extended-spectrum β-lactamase (ESBL)-producing *K. pneumoniae*, can lead to chronic airway inflammation and worsening of lung condition [6]. *K. pneumoniae* is a Gram-negative, capsulated, commensal bacteria that colonizes the skin, respiratory tract, and gastrointestinal tract of humans. As an opportunistic pathogen, it can cause various community-acquired and nosocomial infections, including urinary tract infections (UTIs), respiratory tract infections, and wound or soft tissue infections [7, 8]. The lipopolysaccharide (LPS), capsular polysaccharide (CPS), and biofilm formation of *K. pneumoniae* enable the bacteria to evade the host immune system [9]. Infections caused by *K. pneumoniae* are typically associated with poor prognosis. Even with optimal treatment, pulmonary infections have a mortality rate of 30–50%. The outcome is particularly worse in diabetic, elderly, and immunocompromised patients. Survivors often suffer from long-term lung function impairment, with recovery lasting months [10]. Antibiotics including β-lactams (e.g., third-generation cephalosporins such as cefotaxime and ceftriaxone), aminoglycosides (e.g., gentamicin, amikacin), carbapenems (e.g., meropenem, imipenem/cilastatin) [11] and, quinolones, are utilized in the treatment of *K. pneumoniae* infections, which carry diverse resistance genes due to both chromosomal and plasmid-encoded antibiotic resistance genes (ARGs). Treatment may involve either monotherapy or combination therapy. *K. pneumoniae* represents one of the most significant global contributors to antimicrobial resistance [12]. The escalating use of antibiotics has led to the emergence of ESBL-producing and carbapenem-resistant *K. pneumoniae* strains [13]. The consequences of antibiotic resistance include treatment failures, the need for more expensive and safer alternative medications, and increased morbidity and mortality rates. It also results in prolonged hospital stays and higher healthcare costs. The World Health Organization (WHO) recently recognized antimicrobial resistance as one of the top 10 global public health threats facing humanity. According to reports, ineffective treatment of bacterial infections due to antibiotic resistance causes at least 700,000 deaths annually worldwide. Projections indicate that by 2050, antibiotic-resistant infections could account for up to 10 million deaths each year, with an estimated economic cost of $100 trillion to the global economy [14, 15]. The speed of developing new classes of antibiotics is slower than the emergence of antimicrobial-resistant strains [16]. Therefore, the problems associated with antibiotic resistance and the inability of antibiotics to eliminate biofilm structures have led to the search for new alternative approaches to control pathogens, with phage therapy suggested as a potential solution [17–19].

Bacteriophages are natural antibacterial viruses that specifically target and infect bacteria as intracellular parasites [20, 21]. Indeed, bacteriophages are the potential agents to be utilized in diverse biotechnological applications, varying from food-animal agriculture, human and veterinary medicine to environmental science [22]. The application of bacteriophages in the treatment of bacterial infections has more advantages compared with conventional antibiotics. For example, it does not require sequential administration, it is not harmful for normal flora, it can be carried out with precise targeting of a specific bacterial pathogen, and self-replication at the site of infection is considered a bright side of phages [23]. Bacteriophages coexist with the bacterial communities in the gastrointestinal tract and are known to play a significant role in restoring intestinal eubiosis by eliminating pathogenic strains [24]. In addition, it has been reported that phages can produce depolymerases capable of degrading the biofilm exopolysaccharide matrix [25].

In this study, a novel virulent bacteriophage was isolated from Mofid Children’s Hospital untreated sewage against MDR *K. pneumoniae* in patients with CF. The isolated phage was morphologically and molecularly characterized, and its host specificity, activity spectrum, and biofilm removal assay were determined.

## Materials and methods

### Isolation of *K. pneumoniae* strains

This study was approved by the local ethics committee (Alzahra University) (IR.ALZAHRA.REC.1401.015). All steps of identification and confirmation of bacteria have been carried out in previous studies with the research ethics committees of Shahed University (IR.SHAHED.REC.1400.181) [26, 27], and these steps in summary include: *K. pneumoniae* bacteria were isolated from the sputum of CF patients. All standard biochemical tests were used for the identification of bacteria. Their antibiotic resistance susceptibility was determined by disk diffusion. Sequencing of the 16S rRNA gene was performed. The 16S rRNA gene sequence was amplified nested-PCR by using primers 341f (5´-GC-clamp-ACTCCTACGGGAGGCAGCAG-3´) containing a 40 bp GC-clamp, and 782r (5’-GCGTGGACTACCAGGGTATC-3’). Informed consent was obtained from all participants and/or their legal guardians under the supervision of their physicians to use their clinical samples, and all experiments conformed to the principles set out in the Declaration of Helsinki. Ten clinical isolates of *K. pneumoniae* were used and cultured on nutrient agar (NA) medium (Merck, Germany) and incubated for 24 h at 37 °C. Next, one colony was selected and cultured on MacConkey agar (Merck, Germany) as a differential medium. Gram staining and microscopic analysis were performed to confirm the purification of the *K. pneumonia*e strains. Finally, *K. pneumoniae* 5 was used for phage isolation and amplification due to its high antibiotic resistance compared to other isolates.

### Bacteriophage isolation, purification and amplification

The untreated sewage sample from Mofid Children’s Hospital was collected in September 2020 in Tehran. Next, 40 ml of this sample was centrifuged (Hettich, Germany) at 8000 rpm for 10 min, and the supernatant was filtered using 0.45-µm pore size filters (Bio, China). Then, 5 ml of the filtered supernatant and 5 ml of early log-phase *K. pneumoniae* isolate 5 cultures (1.5 x $\:{10}^{8}$CFU/ml) were added to 5 ml of 10X nutrient broth (Gerhardt, Germany) and placed in an incubated shaker (150 rpm) overnight at 37 °C. (Gerhardt, Germany). After an overnight incubation, centrifugation (8000 rpm, 10 min) was carried out to remove bacterial cells, and the supernatant was filtered using a 0.45 μm pore size filter. To confirm the presence of bacteriophage, 100 µl of the host cells was added to 3 ml of soft nutrient agar (0.7% agar) at 45 °C, and the mixture was poured onto a nutrient agar plate. Then, 10 µl of the supernatant was spotted on four sides of the double-layer agar and incubated at 37 °C (Memmer, Germany) for 24 h. A double agar layer plaque assay technique was used for phage enumeration. 100 µl of phage suspension was serially diluted in phosphate-buffered saline (PBS) (Sigma-Aldrich, USA) and mixed with 500 µl of *K. pneumoniae* isolate 5 (1.5 x $\:{10}^{8}$CFU/ml). Moreover, a sample containing PBS without phage was considered a negative control. Next, it was added to 3 mL of soft nutrient agar (0.7% agar) at 45 °C, and the mixture was poured onto a nutrient agar plate and incubated at 37 °C for 24 h. After incubation, the presence of plaques was observed by comparison with a negative control plate. To purify and propagate the isolated phage, a single plaque was selected, picked, and dissolved in 5 mL of Saline Magnesium (SM) buffer (100 mM NaCl, 50 mM Tris, and 8 mM MgSO₄, pH 7.5) (For each phage enrichment, visually consistent, separate, and distinct plaques were selected). The mixture was then shaken at 50 rpm for 6 h. The phage buffer was transferred into 10 mL of a *K. pneumoniae* culture (1.5 × 10⁸ CFU/mL) and incubated at 37 °C for 24 h. Finally, the phage was stored at 4 °C for further studies [28–30]. For phage storage, 500 µL of the filtered phage solution was thoroughly mixed with 500 µL of 50% glycerol (Merck, Germany) in a sterile cryovial and stored at − 20 °C [31].

### Transmission electron microscopy (TEM)

A high-titer phage suspension (~$\:{10}^{10}$ PFU/ml) was spotted onto a Formvar-carbon-coated copper grid (300 mesh) and fixed with 1% glutaraldehyde (Merck, Germany) in phosphate buffer for 1–3 min. Excess solution was removed using filter paper. The grid was then washed twice with distilled water and negatively stained with 1% uranyl acetate (Sigma-Aldrich, USA) for 1–2 min. Phage morphology was examined using an EM 10 C TEM (Zeiss, Germany) at 100 kV [32, 33].

### Host range determination

The lytic activity of the phage suspension was evaluated against 9 clinical isolates of *K. pneumoniae* (1.5 × 10⁸ CFU/mL), 2 *Pseudomonas aeruginosa* isolates (1.5 × 10⁸ CFU/mL), 2 *Staphylococcus aureus* isolates (1.5 × 10⁸ CFU/mL) from Cystic Fibrosis patients, and one standard strain of *K. pneumoniae* (ATCC 700603) using the spot assay. Clear lytic zones indicated host susceptibility to the phage [34, 35].

### Phage stability tests (temperature, chloroform, pH, and salt stress)

This step was carried out based on previous studies for chloroform [36, 37], pH, temperature [38], and NaCl [39] with some modifications. An equal volume of phage suspension (10⁷ PFU/mL) was mixed with chloroform (Merck, Germany) in two tubes. The first tube was incubated for 24 h and the second tube for 5 h at 37 °C. For thermal stability testing, the phage suspension was added to the nutrient broth (pH 7). Then, it was incubated for 1 h at different temperatures (−20, 4, 37 (control), 50, 60, 70, and 80 °C). For pH stability analysis, nutrient broth was prepared at different pH values ([1, 3, 5, 7, 9]and [11]). Next, the phage suspension was added to the nutrient broth with the desired pH, and the mixtures were incubated for 1 h at 37 °C. To evaluate phage stability in different salt concentrations, the phage suspension was added to nutrient broth containing various concentrations of NaCl (Merck, Germany) (0% (control), 5%, 10%, and 15%), and incubated overnight at 37 °C. Finally, the bacteriophage titer was assessed using the double-layer agar method.

### Multiplicity of infection (MOI)

This test evaluates the phage’s ability to control planktonic bacterial growth at varying multiplicities of infection. For optimal MOI measurement, four test tubes containing bacterial suspension (1.5 × 10⁸ CFU/mL) were prepared. The first tube, without phage, served as a control (MOI 0). The second tube contained 1.5 × 10⁷ PFU/mL (MOI 0.1), the third 1.5 × 10⁸ PFU/mL (MOI 1), and the fourth 1.5 × 10⁹ PFU/mL (MOI 10) of phage suspension. All tubes were incubated (37 °C, 100 RPM), and absorbance at 600 nm was measured at 30-minute intervals for 4 h [30].

### Phage adsorption and one-step growth assay

This step was performed based on a previous study with some modifications [36]. An overnight culture of *K. pneumoniae* was adjusted to 10⁸ CFU/mL in the nutrient broth. Equal volumes of the bacterial culture and phage suspension (10⁷ PFU/mL) were mixed and incubated at 37 °C for 5 and 10 min. The mixtures were centrifuged at 10,000 × g for 5 min and filtered (0.45 μm). The number of free phages was determined using the double-layer agar method, and the reduction in phage titer indicated the number of phages adsorbed to the bacterial cells.

To analyze the phage one-step growth curve, a bacterial culture (1.5 × 10⁸ CFU/mL) was mixed with a phage suspension at an optimal MOI 1. The mixture was incubated for 15 min at 37 °C, then centrifuged (10,000 rpm, 1 min, 4 °C) to remove unabsorbed phages by discarding the supernatant. The pellet containing phage-infected bacterial cells was resuspended in 5 mL of fresh nutrient broth and incubated in a shaker (180 rpm) at 37 °C. Samples were collected every 10 min for 90 min and immediately titrated using the double-layer agar method [38, 40, 41].

### Biofilm assay, degradation and scanning electron microscope (SEM)

The bacterial strains used in this test were *K. pneumoniae* (ATCC 700603) and *K. pneumoniae* 3,5,7. A flat-bottomed 96-well cell culture microtiter plate (SPL Plastic Labware, Korea) was used for the biofilm assay. In this study, 100 µL of Tryptic Soy Broth (TSB) medium (Merck, Germany) supplemented with 0.2% glucose was added to each well, followed by inoculation with 100 µL of a 10⁸ CFU/mL (24-hour *K. pneumoniae* culture) in each well (except the control). It was incubated at 37 °C for 20 h. After incubation, planktonic cells were removed by washing plates three times with pre-warmed (37 °C) physiological saline. The plates were air-dried in a sterile environment for 15 min. Subsequently, 200 µL of TSB supplemented with 0.2% glucose and 50 µL of 0.5% 2,3,5-triphenyl-tetrazolium chloride (TTC) solution (Merck, Germany) were added to each well. The plates were then incubated in darkness at 37 °C with shaking (150 rpm) for 6 h. After this step, absorbance was measured at 405 nm. The test was performed in triplicate for each bacterial strain [42, 43]. For biofilm degradation, following biofilm formation, planktonic cells were removed by washing three times. Then, 50 µL TSB was added to each well. The phage suspension (MOI = 1) was serially diluted (10⁻¹−10⁻¹¹) and added to each well. The last two columns served as controls (medium control and bacterial growth control). Plates were incubated at 37 °C for 24 h. After incubation, plates were washed three times with pre-warmed (37 °C) physiological saline (0.9% NaCl) and dried. Subsequently, 170 µL of TSB supplemented with 0.2% glucose and 30 µL of 1% TTC solution were added to each well. Plates were incubated in darkness in a shaker incubator (150 rpm) at 37 °C for 5 h. Absorbance was then measured at 490 nm. All tests were performed in triplicate for each bacterial strains [42, 44]. Finally for SEM, the clinical isolate of *K. pneumoniae* (1.5 × 10⁸ CFU/mL) treated with phage (MOI = 1) and a control group were prepared according to previous methods with modifications [42, 44]. In this method, samples were placed on coverslips, washed with phosphate-buffered saline, and treated with 4% glutaraldehyde for 2 h at room temperature. Subsequently, samples were placed in alcohol (Tooska-Propyl, Iran) at concentrations of 30%, 50%, 60%, 70%, and 80% for 30 min each, followed by absolute alcohol (100%) for 2 h. After dehydration, samples were placed in a desiccator containing a drying agent. Finally, samples were mounted on SEM stubs using conductive adhesive, sputter-coated with platinum, and visualized by scanning electron microscopy at appropriate magnifications to assess biofilm morphology [45].

### Phage DNA extraction

A high-titer phage suspension (10¹⁰ PFU/ml) of 500 µl was incubated with 1 µl DNase I (2U/µl, Thermo Fisher, USA) and 1 µl RNase I (10U/µl, Thermo Fisher, USA) enzymes for 1 h at 37 °C to digest bacterial nucleic acids. Then, 20 µl EDTA (Merck, Germany) was added and incubated at 75 °C for 15 min. Next, 2 µl proteinase K (Sigma-Aldrich, USA) and 50 µl 10% Sodium Dodecyl Sulfate (SDS) (Scharlau, Spain) were added to the mixture and incubated at 56 °C for 1 h. After this incubation, 375 µl of phenol (Merck, Germany)-chloroform-isoamyl alcohol (Neutron, Iran) solution 25:24:1 (v/v) was added to the phage suspension and centrifuged for 5 min. The upper phase was collected and, 200 µl of chloroform-isoamyl alcohol 24:1 (v/v) was added, followed by centrifugation for 5 min. Subsequently, 40 µl 3 M sodium acetate (Merck, Germany) (pH 5.2) and 800 µl 95% ethanol were added to the solution, which was then incubated at −70 °C for 30 min. After centrifugation for 20 min, 1 ml of 70% ethanol was added to precipitate the DNA, followed by centrifugation for 5 min. Finally, 30 µl sterile deionized water was added to the pellet and stored at −20 °C for further studies [46, 47].

### Genome sequencing and analysis

The bacteriophage DNA genome was sequenced using Illumina high-throughput sequencing (Novogene Company, China). The complete genome sequence of the isolated phage was assembled using CLC Genomics Workbench v.21. The phage termini and packaging strategy were predicted by PhageTerm [48]. The assembled genome was analyzed using Basic Local Alignment Search Tool (BLASTn) against experimental taxonomic nt databases to find the most similar bacteriophage genomes (https://blast.ncbi.nlm.nih.gov/Blast.cgi). The open reading frames (ORFs) were identified using Prokka [49]. The functions of the ORFs were annotated using the protein (BLASTp) from the NCBI server and transmembrane proteins predicted by the TMHMM server [50]. The Promoters were identified using PhagePromoter [51], and tRNAscan [52] was performed to find tRNAs, and the genome structure was visualized in Proksee [53]. The virulence-associated and antibiotic resistance genes were analyzed through the virulence factor database [54] and the comprehensive antimicrobial research database [55].

For determining the relatedness of the phage genome with homologous phages, Easyfig software [56] was used to compare the phage nucleic and amino acid sequences. Phylogenetic analysis of the phage major capsid protein, terminase large subunit, tail fiber genes, and the whole genome phylogenetic tree was performed in default options by using the VIPtree [57], VICTOR web server with 100 bootstrap replicates [58], and NGphylogeny.fr [59].

### Statistical analysis

All data were analyzed and visualized using GraphPad Prism software (version 9) and Microsoft Excel 2019. One-way ANOVA was used to analyze the differences between the phage treatment and the control groups, with a p-value < 0.05 was considered a significant difference. Error bars in the figures represented the standard deviation of the means, and all tests were conducted in three replicate experiments.

## Results

### Antimicrobial susceptibility test report

In previous studies [26, 27], 15 different antibiotics were tested on 10 clinical isolates of *K. pneumoniae*. Table 1 shows the antimicrobial susceptibility test report for *K. pneumoniae* isolates from CF patients. The highest sensitivity was observed for the antibiotic imipenem (78% of the isolates), while the highest resistance was noted for the antibiotic ciprofloxacin (67% of the isolates).

**Table 1:** Patterns of antimicrobial susceptibility test of the *K. pneumoniae* isolates

K. pneumoniae Isolated	Resistant	Intermediate	Sensitive
Isolated 1	FM, NA, CP	CFM	CRO, AN, IPM, GM, CF, SXT
Isolated 2	VAN	FOX, FM	CFM, CP, NA, SXT, CRO, CAZ, AN
Isolated 3	SXT, GM, CAZ, CP, IPM, NOR	–	AN
Isolated 4	NOR, CP, AN, GM	–	CRO, CF, IPM
Isolated 5	AN, CRO, CF, CP, SXT, AM, NOR, CFM, LEVO, VAN, CAZ	FM	IPM, FOX
Isolated 6	-	–	IPM, AN, CF, CRO, CAZ, CP, CFM
Isolated 7	FM, AZM, NOR, CP, NA, GM, AN	–	–
Isolated 8	FM, CF	–	AN, CAZ, FOX, CP, IPM, CFM, SXT, GM, NA, CRO
Isolated 9	AZM	–	AN, CAZ, NOR, CP, GM, IPM
Isolated 10	CP, NA, NOR, GM	–	CRO, CF, IPM, AN, CFM

CFM: cefixime, FM: nitrofurantoin, NA: nalidixic acid, CP: ciprofloxacin,: FOX: cefoxitin, CF: cefalotin, CAZ: cefazidime, AM: ampicillin, GM: gentamicin, AN: amikacin, CRO: ceftriaxone, SXT: trimethoprim Sulfamethoxazole, NOR: norfloxacin, IPM: imipenem, AZM: azithromycin

### Isolation of phage

**Fig. 1:**
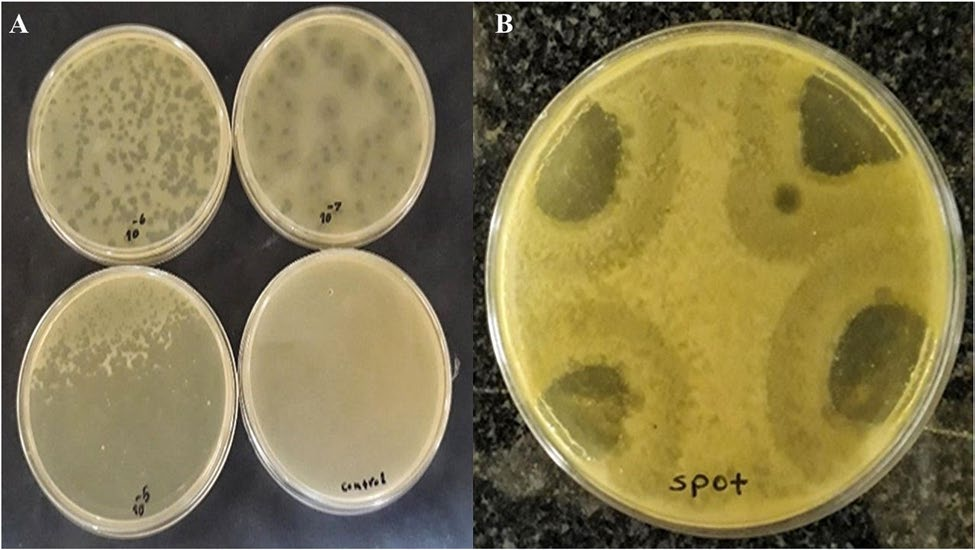
Isolation of the phage by double-layer agar method (**A**) and spot test (**B**). Clear plaques measuring 2–4 mm in diameter appeared on the plates, indicating the lytic activity of the isolated phage. These plaques represent areas where the phage has successfully lysed bacterial cells, confirming its effectiveness in targeting the host. (**A**)

The spot test showed that this isolate is sensitive to the phage, as indicated by the formation of clear zones on the plate (Fig. 1B). The result of double-layer agar showed one phage that formed a transparent round plaque with a diameter of about 2–4 mm on the bacterial lawn of *K. pneumoniae* isolate 5 was isolated from the untreated sewage sample of the hospital (Fig. 1A).

### Phage morphology

**Fig. 2:**
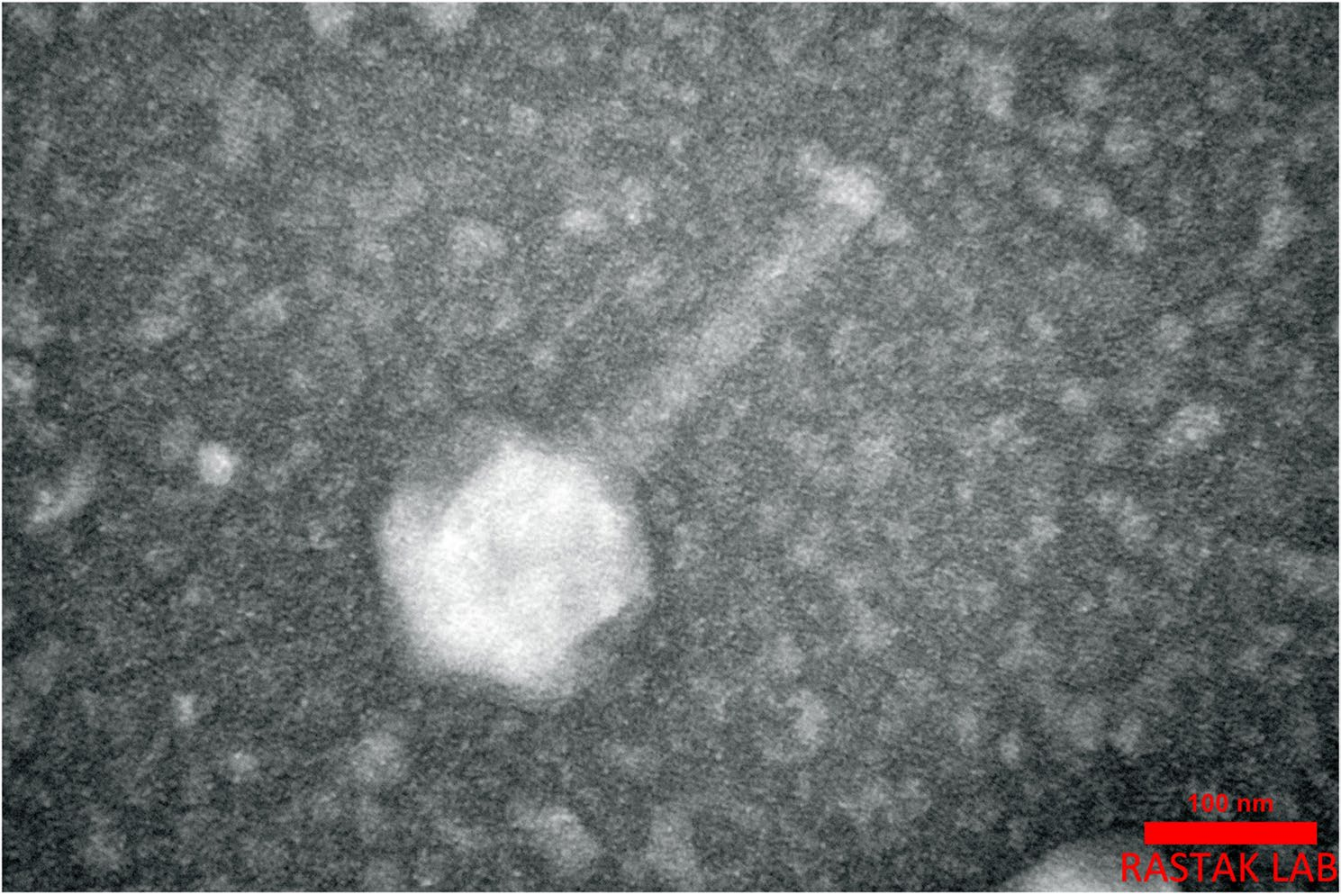
Transmission Electron Micrograph. The TEM micrograph of the AEV23 phage, with an icosahedral head and elongated contractile tail. Scale bar = 100 nm

TEM micrograph analysis of the phage showed that it had a typical icosahedral structure with a contractile tail, featuring a head diameter of approximately 120 nm and a tail length of approximately 171 nm (Fig. 2).

### Determination of the phage host range

The lytic activity of the isolated phage was examined on 14 bacterial isolates. The spot test results showed that the AEV23 phage had no lytic activity against *S. aureus* and *P. aeruginosa* isolates (without any halos or plaques), but it was effective against 7 out of 9 isolates of *K. pneumoniae*, including the standard *K. pneumoniae* strain. The large, clear plaques formed in the bacterial lawns also indicated their strong lytic effect.

### Stability of the phage particles to temperature, chloroform, pH, and salt stress

**Fig. 3:**
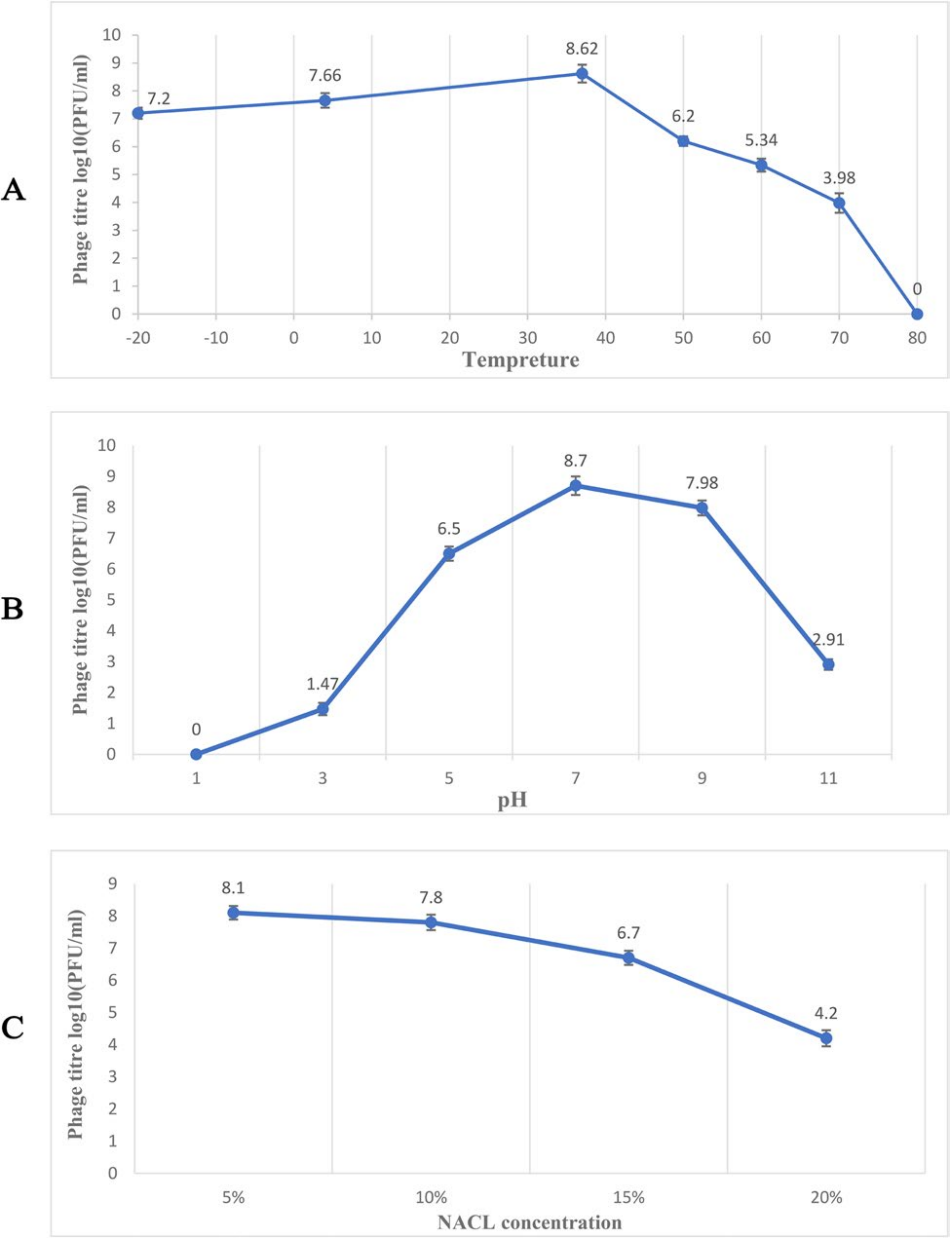
Stability of the phage to temperature, pH, and salt stress tests. (**A**) Thermal, the best phage activity, which was examined in the temperature range of −20 °C to 80 °C, was observed at 37 °C, while at 80 °C, its activity dropped to zero. (**B**) For pH, the best activity was observed at pH 7, and no activity was detected at pH 1. (**C**) In the NaCl stability tests of the phage, it was found that as the salt concentration increased, phage activity decreased. The Bars represent the mean ± SD from the triplicate experiments

The best phage activity was observed at 37 °C, and it exhibited appropriate activity in the temperature range of −20 °C to 40 °C. However, its lytic activity decreased as the temperature increased. Specifically, the phage titer decreased by 2 logs at 50 °C, 3 logs at 60 °C, 4 logs at 70 °C, and at 80 °C, phage activity decreased completely (Fig. 3A). The isolated phage demonstrated good activity at a pH between 4 and 9, with the best activity at pH 7; however, it did not show any activity at pH 1. Additionally, the phage titer decreased by 6 logs at pH 11 (Fig. 3B), indicating that the stability of this phage notably decreased in environments with high pH. In the chloroform stability test, phage activity did not decrease after 5 and 24 h. The results also showed that the phage had good activity at salt concentrations of 5% and 10%, but phage activity decreased as the salt concentration increased (Fig. 3C).

### Optimal multiplicity of infection (MOI)

**Fig. 4:**
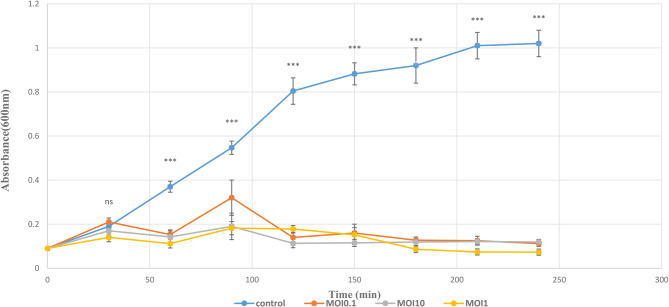
MOIs of AEV23 phage (0.1, 1 and 10) for *K. pneumoniae*. The phage exhibited similar activity at MOIs of 0.1, 1, and 10, but the highest progeny was observed at MOI = 1. Data are shown as the mean ± SD from the triplicate experiments. The asterisks (***P < 0.001, **P < 0.01 or *P < 0.05) indicate a significant difference between each phage-treated group and the control with no phage treatment and, the ns shows non-significant difference

The results indicated that all tested MOIs were similarly effective against the host isolate. The highest phage progeny was observed at MOI = 1 (OD₆₀₀ = 0.073), while MOI = 10 (OD₆₀₀ = 0.113) showed results that were relatively similar to MOI = 1. The least progeny was observed at MOI = 0.1 (Fig. 4).

### Phage adsorption and one step growth

**Fig. 5:**
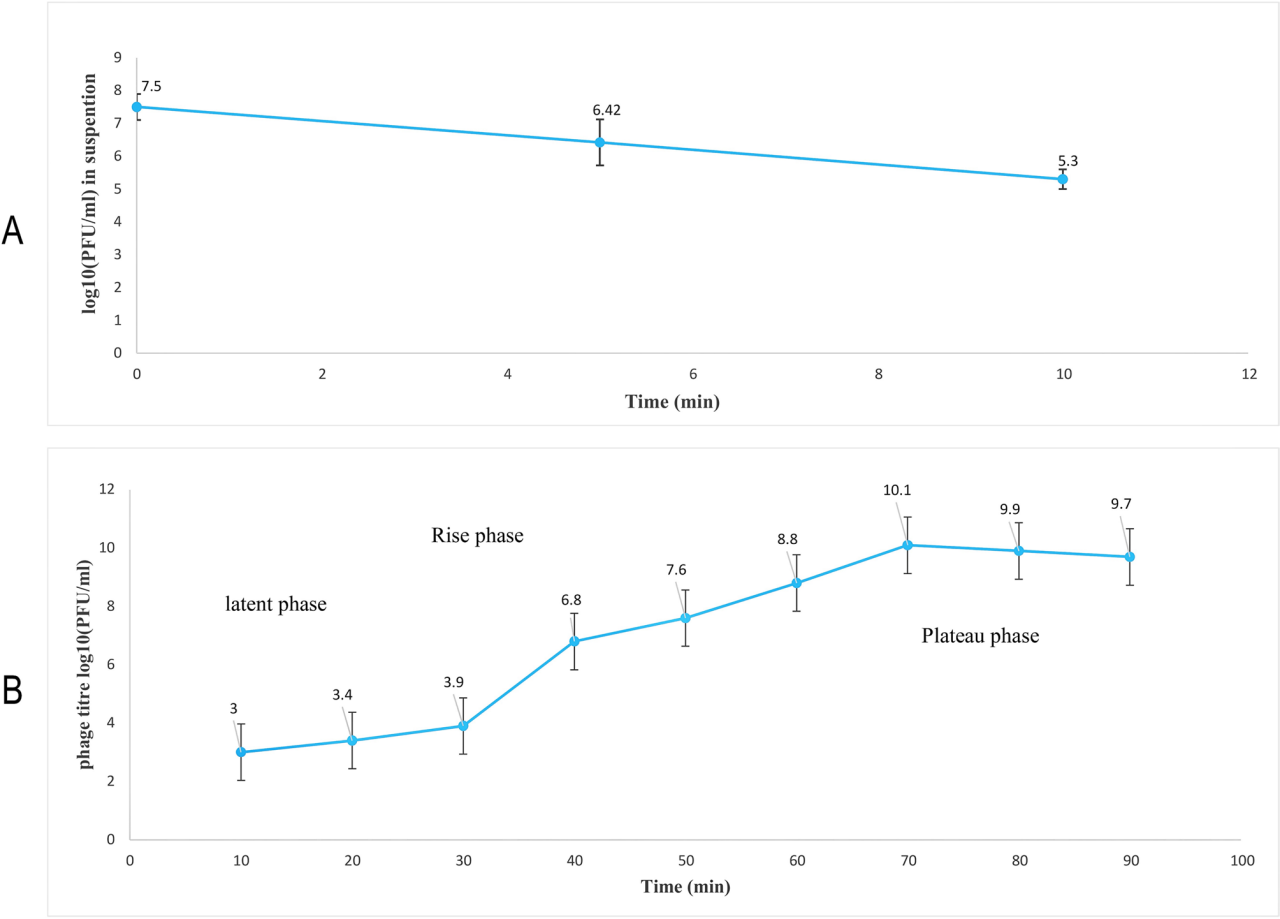
Adsorption (**A**) and One-step growth curve of phage (**B**) in *K. pneumoniae*. (**A**) The absorbed bacteriophage titration showed that 92% of the isolated phages were absorbed by bacterial cells in 5 min. (**B**) The one-step growth experiment revealed a latent period of 30 min and a rise period of 40 min. The burst size was approximately 98 phage particles per infected bacterium. The bars represent the mean ± SD from the triplicate experiments

The adsorbed bacteriophage titration was evaluated using the double-layer method. The percentages of phage adsorption at two time points were plotted: 92% of the isolated phages were adsorbed to bacterial cells in 5 min. (Fig. 5 A). The one-step growth experiment was performed to determine the latent period and burst size of the phage. The latent period, log or rise period, and plateau period are indicated in Fig. 5B The latent period and rise period were 30 min and 40 min, respectively. A growth plateau started at 70 min, and the burst size was approximately 98 phage particles per infected bacterium (Fig. 5B).

### Biofilm degradation assay and scanning electron microscope (SEM)

**Fig. 6:**
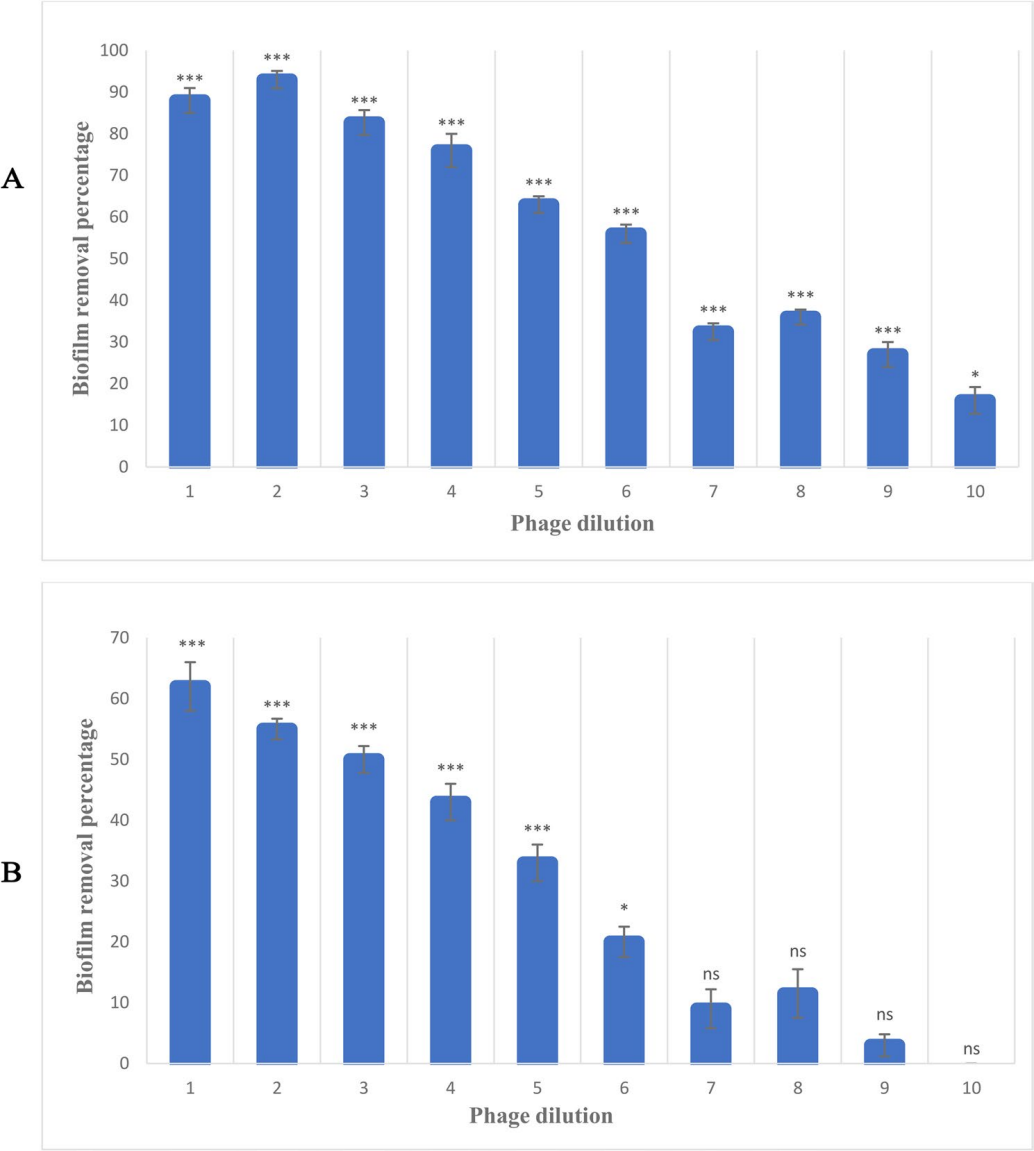
Phage biofilm removal assay. (**A**) The highest biofilm removal of *K. pneumoniae* isolate 5 was observed at a dilution of $\:{10}^{-2}$ (**B**) The highest biofilm removal of *K. pneumoniae* standard strain was observed at a dilution of $\:{10}^{-1}$. Data are shown as the mean ± SD from the triplicate experiments. The asterisks (***P < 0.001, **P < 0.01 or *P < 0.05) indicate a significant difference between each phage-treated group and the control with no phage treatment, and the ns shows a non-significant difference

The results demonstrated that *K. pneumoniae* isolate 5 produced more biofilm compared to the other isolates; therefore, it was chosen for the determination of the phage biofilm removal test. According to our results, the reduction in the absorbance of each well can indicate the effectiveness of the phage in destroying the bacterial biofilm. Consequently, the percentage of biofilm removal capability of the isolated phage was calculated separately for *K. pneumoniae* isolates. The absorption rate of the wells decreased in samples treated with different dilutions of phage compared to the control group. Phage dilution $\:{10}^{-2}$ had the highest ability to destroy the bacterial biofilm (Table 2) (Fig. 6 A). Moreover, the absorption rate was also calculated for the standard strain. The results showed that the absorption rate was reduced in the treated group, with the highest percentage of biofilm removal capability related to the phage dilution $\:{10}^{-1\:}$(Table 2) (Fig. 6B).

**Table 2:** The biofilm removal ability of the phage

Standard strain	K. pneumoniae isolates of CF patients	Phage dilution
62%	88%	$\:{10}^{-1}$
55%	93%	$\:{10}^{-2}$
50%	82.7%	$\:{10}^{-3}$
43%	76%	$\:{10}^{-4}$
33%	63%	$\:{10}^{-5}$
20%	56%	$\:{10}^{-6}$
9%	32.5%	$\:{10}^{-7}$
11.5%	36%	$\:{10}^{-8}$
3%	27%	$\:{10}^{-9}$
Effectless	16%	$\:{10}^{-10}$

**Fig. 7:**
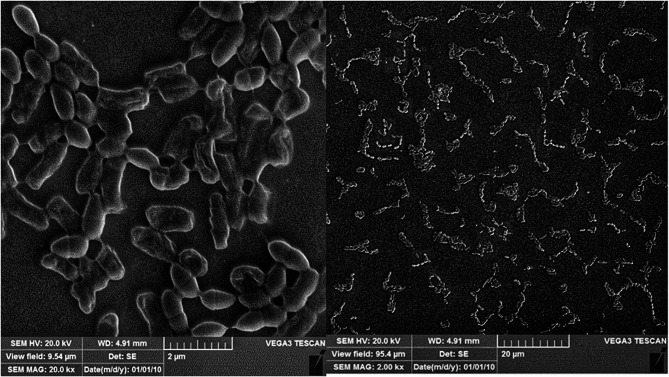
SEM micrograph of *K. pneumoniae* biofilm formation. *K. pneumoniae* formed biofilms on the cover glass. The scale of the left image is 2 μm, and that of the right image is 20 μm

SEM micrographs show the formation of *K. pneumoniae* biofilms, and Fig. 7 illustrates the compact, dense biofilms that formed on the coverslip.

**Fig. 8:**
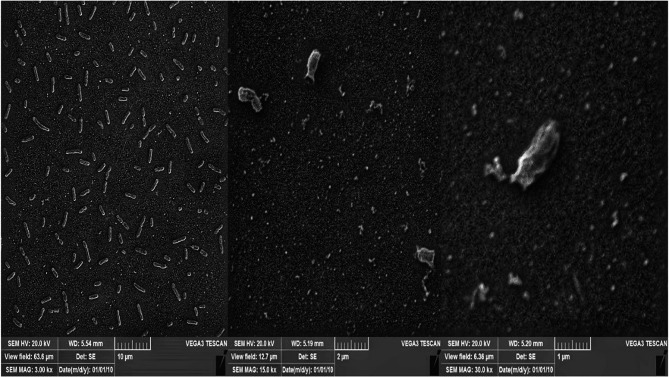
SEM micrograph of biofilm destruction by phage. The phages could destroy the biofilm formed by *K. pneumoniae.* The scale of the left image is 10 μm, the middle image is 2 μm, and the right image is 1 μm

After treatment with bacteriophage, adherent *K. pneumoniae* cells were significantly reduced compared to the control group. The phage-treated surfaces were heavily colonized. Exposure to phages resulted in a significant reduction in biofilm cell density, with only a few cells remaining visible, confirming the results of our biofilm removal experiment (Fig. 8).

### Genomic analysis of phage AEV23

**Fig. 9:**
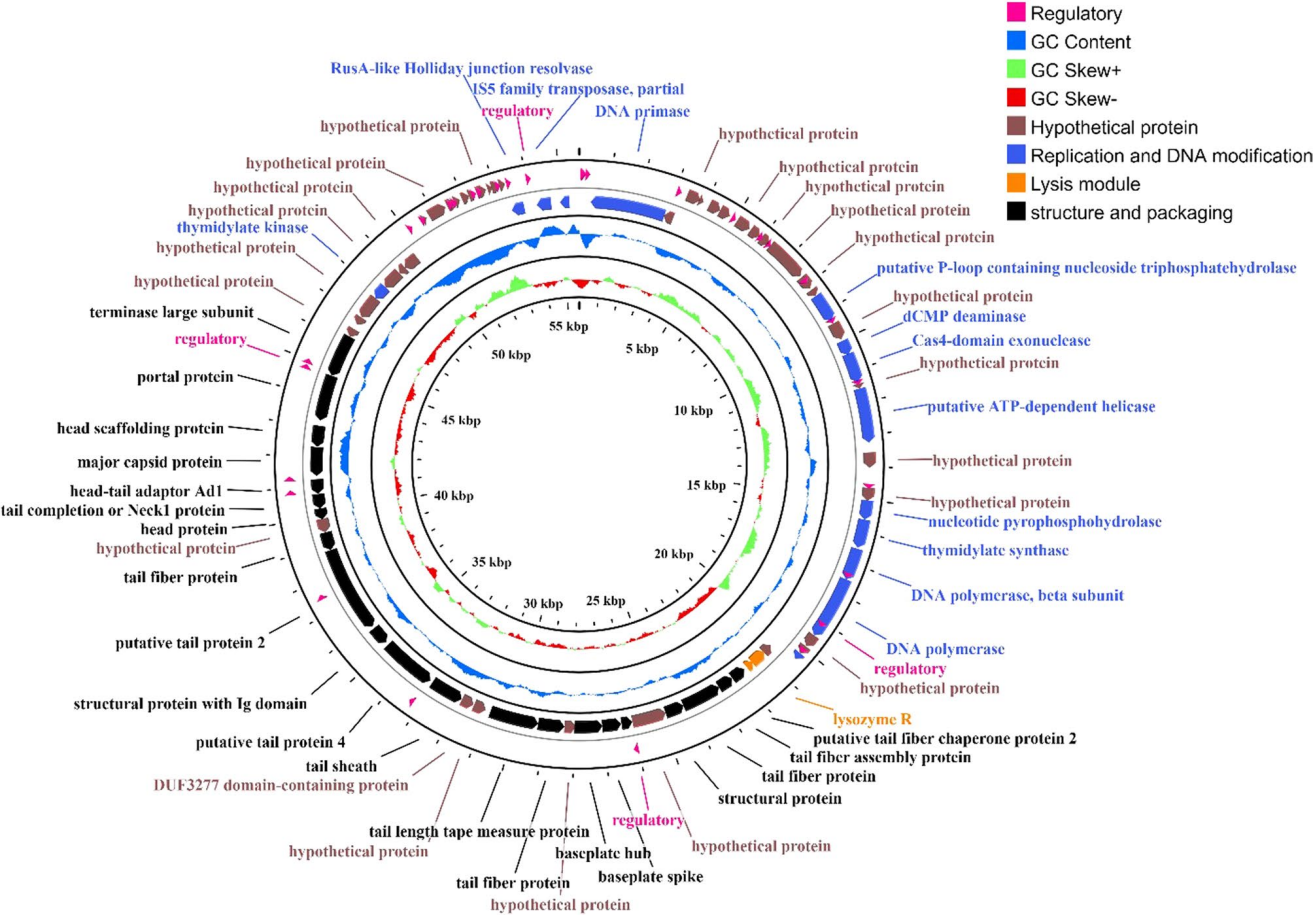
Circular map of the *Klebsiella* phage VB_KpM-AEV23 genome. The innermost circle represents the GC skew (G − C)/(G + C). Outward indicates > 0, and inward indicates < 0. The blue circles in the middle represent the GC content (outward indicates greater than the average GC content compared to the whole genome, and inward indicates the opposite). The outermost circles represent ORFs encoded in the genome, with different colors representing different functions

Phage AEV23 has a linear and double-stranded DNA with a length of 55,637 bp, a GC content of 45.9%, and a direct terminal repeat region of 3,915 bp. A total of seventy-six putative Open Reading Frames (ORFs) were predicted (Table 3), including 38 functional genes, 22 structural and packaging genes, 14 DNA replication and modification genes, 2 lysis modules, and the remaining ORFs are hypothetical genes. Additionally, thirty-one regulatory elements were annotated (Fig. 9).

**Table 3:** Blast predicted ORFs annotation results

ORF number	Representative Sequence	Related species	Identity%	Query coverage%	Protein length	E value	Total score	Accession number
ORF01	DNA primase	Salmonella phage SE4	93.7	100	844	0	1666	YP_009845582.1
ORF02	Hypothetical protein	Escherichia coli	92.7	100	110	6.85E-71	213	WP_166428434.1
ORF03	Hypothetical protein HOV36_gp54	Salmonella phage ZCSE2	100	100	141	2.37E-102	295	YP_009821769.1
ORF04	Hypothetical protein HOV36_gp53	Salmonella phage ZCSE2	93.3	100	30	3.44E-12	58.9	YP_009821768.1
ORF05	Hypothetical protein HOV36_gp51	Salmonella phage ZCSE2	98.4	100	122	9.41E-87	253	YP_009821766.1
ORF06	Hypothetical protein HWC20_gp07	Salmonella phage SE4	90.3	100	113	1.70E-71	214	YP_009845587.1
ORF07	Hypothetical protein	Salmonella phage S144	100	95	150	1.13E-99	288	QMV47886.1
ORF08	Hypothetical protein HOV36_gp48	Salmonella phage ZCSE2	99.1	100	110	4.11E-77	228	YP_009821763.1
ORF09	Hypothetical protein HWC20_gp12	Salmonella phage SE4	94.9	100	98	7.94E-65	196	YP_009845592.1
ORF10	Hypothetical protein	Salmonella phage S144	95.4	100	437	0	877	QMV47883.1
ORF11	Hypothetical protein HWC20_gp14	Salmonella phage SE4	95.7	100	141	1.28E-98	285	YP_009845594.1
ORF12	Hypothetical protein HOV36_gp44	Salmonella phage ZCSE2	97.4	100	76	7.37E-50	157	YP_009821759.1
ORF13	Putative P-loop containing nucleoside triphosphatehydrolase	Salmonella phage S144	99.7	100	296	0	615	QMV47880.1
ORF14	Hypothetical protein HOV36_gp42	Salmonella phage ZCSE2	97.8	100	179	6.19E-128	362	YP_009821757.1
ORF15	dCMP deaminase	Salmonella phage SE4	91.6	100	154	2.31E-104	301	YP_009845598.1
ORF16	Cas4-domain exonuclease	Salmonella phage SE4	94.8	96	303	0	591	YP_009845599.1
ORF17	Hypothetical protein HOV36_gp39	Salmonella phage ZCSE2	100	100	49	5.88E-28	99.8	YP_009821754.1
ORF18	Putative ATP-dependent helicase	Salmonella phage S144	99.6	100	557	0	1158	QMV47876.1
ORF19	Hypothetical protein HOV36_gp36	Salmonella phage ZCSE2	100	100	156	2.11E-104	301	YP_009821751.1
ORF20	Hypothetical protein	Salmonella phage S144	99.2	100	132	3.41E-94	273	QMV47870.1
ORF21	Nucleotide pyrophosphohydrolase	Salmonella phage SE4	94.4	100	197	1.65E-140	395	YP_009845605.1
ORF22	Thymidylate synthase	Salmonella phage ZCSE2	100	100	294	0	617	YP_009821747.1
ORF23	DNA polymerase, beta subunit	Salmonella phage SE4	96.4	100	332	0	669	YP_009845607.1
ORF24	DNA polymerase	Salmonella phage SE4	95.4	100	656	0	1313	YP_009845608.1
ORF25	Hypothetical protein bas20_0026	Escherichia phage FritzHoffmann	64.4	65	133	1.59E-29	110	QXV79291.1
ORF26	Hypothetical protein HOV36_gp29	Salmonella phage ZCSE2	98.9	100	91	2.15E-60	184	YP_009821744.1
ORF27	DksA-like zinc-finger protein	Salmonella phage ZCSE2	98.6	100	74	1.78E-48	153	YP_009821743.1
ORF28	Hypothetical protein HOV36_gp27	Salmonella phage ZCSE2	100	100	109	1.75E-73	219	YP_009821742.1
ORF29	Lysozyme R	Escherichia phage vB_EcoM_fHy-Eco03	90.7	102	180	1.39E-123	352	QTD79406.1
ORF30	Holin	Salmonella phage ZCSE2	100	100	87	9.92E-58	177	YP_009821740.1
ORF31	Putative tail fiber chaperone protein 2	Salmonella phage S144	97.6	100	169	6.65E-122	346	QMV47859.1
ORF32	Tail fiber assembly protein	Salmonella phage ZCSE2	99.4	100	180	1.77E-132	374	YP_009821738.1
ORF33	Tail fiber protein	Salmonella phage SE4	91.1	100	449	0	852	YP_009845617.1
ORF34	Structural protein	Salmonella phage SE4	96.8	100	217	5.50E-153	429	YP_009845618.1
ORF35	Hypothetical protein HWC20_gp39	Salmonella phage SE4	97.2	100	387	0	769	YP_009845619.1
ORF36	Baseplate wedge subunit	Salmonella phage SE4	90.8	100	119	2.33E-78	232	YP_009845620.1
ORF37	Baseplate spike	Salmonella phage SE4	90.7	100	214	1.49E-124	357	YP_009845621.1
ORF38	Baseplate hub	Salmonella phage ZCSE2	99.4	100	316	0	654	YP_009821732.1
ORF39	Hypothetical protein HWC20_gp43	Salmonella phage SE4	99.2	100	130	3.09E-91	266	YP_009845623.1
ORF40	Tail fiber protein	Salmonella phage SE4	98.7	100	298	0	598	YP_009845624.1
ORF41	Tail length tape measure protein	Salmonella phage SE4	96.9	100	553	0	1083	YP_009845625.1
ORF42	Hypothetical protein HWC20_gp46	Salmonella phage SE4	98.6	100	142	4.14E-97	281	YP_009845626.1
ORF43	DUF3277 domain-containing protein	Escherichia coli	96.5	100	142	2.72E-98	285	WP_166428470.1
ORF44	Tail sheath	Salmonella phage SE4	96.6	100	379	0	768	YP_009845628.1
ORF45	Putative tail protein 4	Salmonella phage S144	98.6	100	624	0	1189	QMV47845.1
ORF46	Structural protein with Ig domain	Salmonella phage ZCSE2	99.5	100	207	4.17E-140	395	YP_009821724.1
ORF47	Putative tail protein 2	Salmonella phage S144	99.7	99	985	0	2028	QMV47843.1
ORF48	Tail fiber protein	Salmonella phage ZCSE2	99.5	100	206	1.41E-146	412	YP_009821722.1
ORF49	Hypothetical protein HOV36_gp06	Salmonella phage ZCSE2	100	100	158	2.63E-113	323	YP_009821721.1
ORF50	Head protein	Salmonella phage SE4	97.6	100	125	9.72E-89	259	YP_009845634.1
ORF51	Tail completion or Neck1 protein	Salmonella phage SE4	95.1	100	162	6.21E-110	315	YP_009845635.1
ORF52	Head-tail adaptor Ad1	Salmonella phage SE4	94	100	166	4.57E-114	326	YP_009845636.1
ORF53	Major capsid protein	Salmonella phage vB_SenM_PA13076	93.8	100	325	0	607	ATI16159.1
ORF54	Head scaffolding protein	Salmonella phage ZCSE2	100	100	238	7.53E-174	483	YP_009821793.1
ORF55	Portal protein	Salmonella phage SE4	91.9	100	520	0	1005	YP_009845639.1
ORF56	Terminase large subunit	Salmonella phage vB_SenM_PA13076	95.4	100	482	0	974	ATI16162.1
ORF57	Hypothetical protein HOV36_gp75	Salmonella phage ZCSE2	99	100	98	5.40E-65	197	YP_009821790.1
ORF58	Hypothetical protein HWC20_gp62	Salmonella phage SE4	89.5	100	95	3.11E-59	182	YP_009845642.1
ORF59	Hypothetical protein	Salmonella phage S144	99.6	100	267	0	547	QMV47831.1
ORF60	Thymidylate kinase	Salmonella phage SE4	96.4	100	166	2.59E-117	334	YP_009845644.1
ORF61	Hypothetical protein HWC20_gp65	Salmonella phage SE4	89.7	100	223	4.34E-150	422	YP_009845645.1
ORF62	Hypothetical protein	Salmonella phage S144	98.9	100	89	4.53E-60	184	QMV47828.1
ORF63	Hypothetical protein HWC20_gp67	Salmonella phage SE4	97	95	178	2.34E-120	343	YP_009845647.1
ORF64	Hypothetical protein	Escherichia coli	95.9	100	49	7.65E-28	99.8	WP_166428490.1
ORF65	Hypothetical protein HWC20_gp69	Salmonella phage SE4	94.1	100	188	1.27E-129	367	YP_009845649.1
ORF66	Hypothetical protein HBKIJOIA_00063	Salmonella phage S1	100	100	87	7.22E-58	178	UOK16664.1
ORF67	Hypothetical protein HWC20_gp71	Salmonella phage SE4	98.2	92	62	2.57E-31	108	YP_009845651.1
ORF68	Hypothetical protein HOV36_gp64	Salmonella phage ZCSE2	98.7	100	78	3.74E-52	163	YP_009821779.1
ORF69	Hypothetical protein HOV36_gp63	Salmonella phage ZCSE2	100	100	66	4.59E-42	136	YP_009821778.1
ORF70	Hypothetical protein HWC20_gp74	Salmonella phage SE4	90.1	100	101	2.47E-63	193	YP_009845654.1
ORF71	Hypothetical protein	Salmonella phage S144	97.5	100	40	1.34E-19	78.2	QMV47897.1
ORF72	Hypothetical protein HOV36_gp60	Salmonella phage ZCSE2	98.8	100	81	7.74E-55	170	YP_009821775.1
ORF73	Hypothetical protein HOV36_gp58	Salmonella phage ZCSE2	98.4	100	62	1.13E-38	127	YP_009821773.1
ORF74	RusA-like Holliday junction resolvase	Salmonella phage SE4	98.6	100	141	8.36E-101	291	YP_009845581.1
ORF75	IS5 family transposase, partial	Escherichia coli	90.8	94	161	3.35E-102	299	WP_241342168.1
ORF76	Methyltransferase	Uncultured Mediterranean phage	35.7	69	102	2.51E-04	45.4	ANS04953.1

Furthermore, the absence of integrase, tRNA, lysogenic, antibiotic resistance, and virulence factor genes indicates that phage AEV23 is a virulent phage targeting *K. pneumoniae*.

### Phylogenetic tree and comparative genomic analysis

**Fig. 10:**
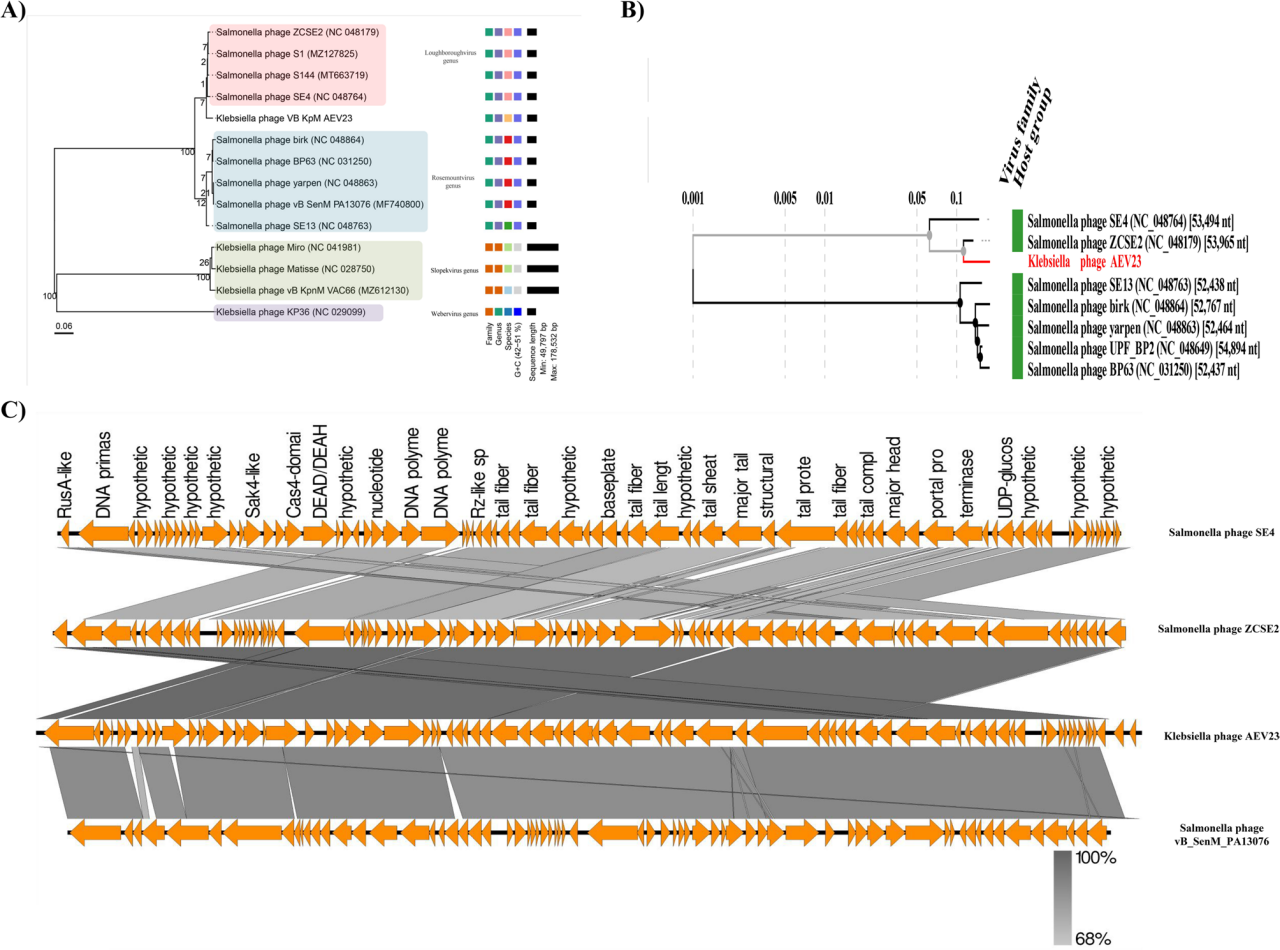
Whole genome phylogenetic tree and genomic comparison. (**A**) The whole genome nucleotide phylogenetic tree of phage AEV23 and related phages, constructed by VICTOR, revealed that this phage is related to the *Loughboroughvirus* genus. The legend on the right side of the tree indicates the family, genus, and species, with squares of the same color representing organisms belonging to the same family, genus, or species. Numbers near the nodes represent bootstrap values. (**B**) Proteomic tree from viptree server. Branch lengths are logarithmically scaled from the root of the entire proteomic tree. (**C**) Comparison of the draft genome sequence of phage AEV23 with three other homologous phages using EasyFig v2.2.5. The arrows of different colors illustrate predicted ORFs and the direction of transcription. The color intensity, ranging from gray to black, indicates the level of amino acid sequence identity (68–100%)

BLASTn analysis showed that the genome of phage AEV23 has the highest sequence similarity to *Salmonella* phage S1 (coverage: 95%, identity: 96.4%), *Salmonella* phage ZCSE2 (coverage: 95%, identity: 96.25%), and *Salmonella* phage S144 (coverage: 95%, identity: 97.2%), all of which belong to the *Loughboroughvirus* genus. Whole genome phylogeny of the phage was carried out using VICTOR (nucleotide) and VIPtree (proteomic), which showed that *Salmonella* phage ZCSE2 and *Salmonella* phage SE4 (*Loughboroughvirus* genus) were the closest phages. Additionally, *Salmonella* phage SE13, Brik, Yarpen, UPF_BP2, and BP63 (*Rosemountvirus* genus) were matched to AEV23 with more distant similarity (Fig. 10 A and B). The nucleotide-based whole genome tree revealed that phage AEV23 is in a separate branch related to the *Loughboroughvirus* genus. Moreover, comparative genomic analysis was performed with similar phages to analyze the genomic evolutionary relationships (Fig. 10 C).

**Fig. 11:**
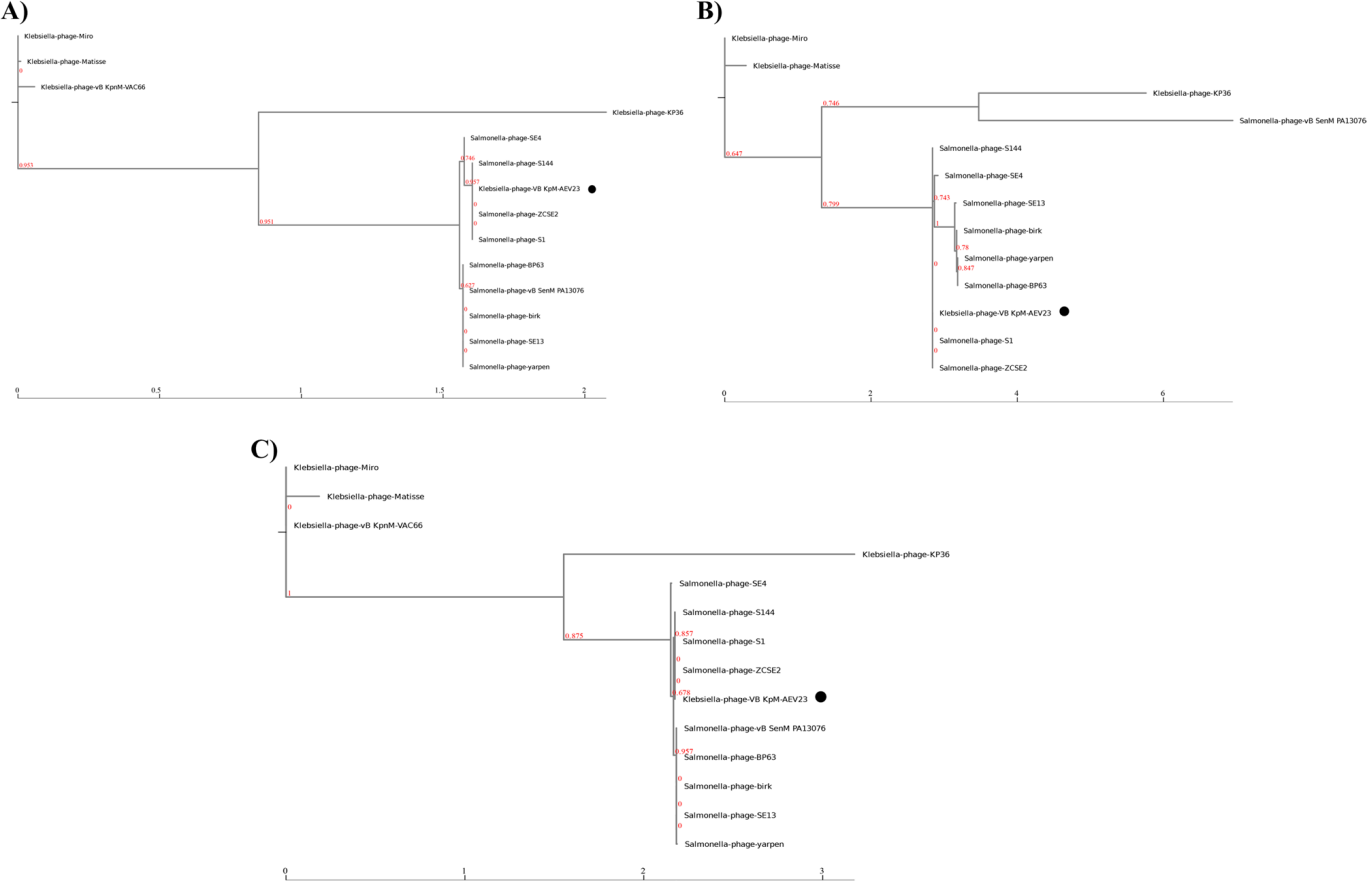
Protein phylogenetic tree of phage AEV23. The figure shows the protein phylogenetic tree of phage AEV23 and 13 other phages based on the deduced amino acid sequences of (**A**) major capsid protein, (**B**) tail fiber protein, and (**C**) terminase large subunit. The values on the nodes of the branches indicate the branch lengths. The position of phage AEV23 in the figure is marked with a black circle

The results indicated that AEV23 is more closely related to phage ZCSE2 than to other related phages. There is a genomic rearrangement at the 5’ end of phage AEV23, including the packaging and DNA modification modules. To better understand the phage’s relationship and taxonomic position, the major capsid protein (ORF53), terminase large subunit (ORF56), and tail fiber (ORF33) genes were chosen for phylogenetic analysis. Three genes had similar phylogenic results, indicating that phage AEV23 belongs to the *Loughboroughvirus* genus (Fig. 11).

## Discussion

MDR *K. pneumoniae* infections are a major healthcare challenge. These strains utilize mechanisms such as extended-spectrum β-lactamase and carbapenemase production [60]. Biofilm formation reduces antibiotic effectiveness and protects bacteria from host responses [61].

Therapeutic options are limited, requiring alternative strategies to address antibiotic resistance. Bacteriophages, with their high specificity and antibacterial properties, are promising candidates [62]. Thus, phage therapy is suggested for inhibiting the growth of *Klebsiella* strains [47]. In this study, A novel phage, AEV23 was isolated from untreated sewage at Mofid Children’s Hospital in Tehran, Iran, and its molecular and physiological characterizations were determined. The isolated phage formed clear plaques measuring 2–4 mm in diameter, which indicates a fully lytic (virulent) phenotype. This characteristic is essential for therapeutic applications to avoid the risks associated with lysogeny. When bacterial growth reaches a plateau, phage replication slows or stops, preventing further plaque expansion [63]. Hazy halo zones suggested the ability of this phage to produce polysaccharide-degrading enzymes [19, 64]. Previous studies have shown that phage polysaccharide depolymerases can degrade bacterial capsules [65, 66]. AEV23 showed high specificity, infecting only *K. pneumoniae*, and strong lytic activity, making it a potential candidate for phage therapy in combination with other phages as a cocktail [67]. Moreover, phages used in therapy must be strictly lytic to avoid transferring virulent bacterial genes, this characteristic is essential [68]. Our study confirmed previous findings that phages are highly specific to their host cell receptors [69]. Temperature and pH are critical factors influencing phage viability and stability [19]. The studied phage showed optimal activity at 37 °C, remained stable at 4 °C and − 20 °C, but its stability declined at 70 °C and was completely lost at 80 °C due to protein denaturation [70]. It also exhibited optimal activity at pH 7, remained active between pH 4 and 9, but lost activity at pH 1 and was significantly reduced at pH 11, indicating sensitivity to extreme acidic and alkaline conditions that affect capsid integrity and receptor binding [71]. These findings align with those of Jamal et al., who reported that phage Z remained stable between 37 °C and 70 °C and at pH 5 to 9. AEV23 also showed strong cold tolerance at − 20 °C, supporting its clinical storage and transport potential [33]. In another study, the *K. pneumoniae* phage vB_KleS-HSE3 demonstrated stability for 30 minutes across pH 5 to 11 and temperatures below 70 °C [40]. Tailed bacteriophages lack lipids but about one-third are chloroform-sensitive [37, 72]. The isolated phage was chloroform-resistant, consistent with findings that most tailed phages show such resistance [37]. It maintained good activity at 5% and 10% NaCl, though activity declined with higher concentrations. Stability tests confirmed strong environmental tolerance. However, Komijani et al. reported a phage that tolerated up to 18% NaCl after 24 hours [39]. Determining MOI is essential for maximizing phage production [73]. This study tested MOIs of 0.1, 1, and 10, finding reduced absorption rates across all compared to the control. MOI 1 had the most significant impact on host cells. While higher MOIs can accelerate bacterial clearance, excessively high levels may trigger lysis inhibition and reduce phage replication [74]. The highest phage yield at MOI 1, with reduced efficiency at MOI 10, indicates that a moderate MOI is optimal for balancing replication and bacterial elimination. The absorption test showed that 92% of phages attached to *K. pneumoniae* within 5 minutes, indicating strong affinity. This rapid adsorption is critical for effective phage therapy. Adsorption time can vary based on bacterial receptors and environmental conditions [75]. These findings align with Kęsik-Szeloch et al., who reported that 5 out of 32 phages isolated in Poland adsorbed to *K. pneumoniae* within 5 minutes at rates above 98% [37]. The phage growth curve showed 30 minutes latent period and a burst size of about 98 virions per cell, with replication stabilizing at 70 minutes, marking the end of the lytic cycle. These parameters are crucial for understanding phage-host interactions in therapeutic contexts, where controlled replication is important [75]. Variations in these values across studies may relate to phage size and envelope structure [76]. The short incubation time, high absorption rate, and large burst size suggest this phage is a strong candidate for phage therapy [77]. Phage isolation targeting biofilms is crucial, especially for CF patients where airway infections are biofilm-associated [78]. Phages show varying biofilm removal abilities at different dilutions due to receptor affinity differences [45]. In this study, AEV23 reduced *K. pneumoniae* biofilms by ~ 93%, indicating strong anti-biofilm potential. Phage depolymerase enzymes aid in EPS degradation, biofilm penetration, and bacterial lysis [79]. Previous studies reported biofilm reductions of 76% by phage PG14 (2022) [80] and 50–66% by phage Z (2015) [33]. AEV23’s effectiveness underscores the therapeutic value of phages in treating biofilm-related infections and reducing antibiotic dependence. The comparative and phylogenetic analysis showed that phage AEV23 belongs to the *Loughboroughvirus* genus in the *Caudoviricetes* class. The receptor binding proteins (RBPs) of the phages in the tail fibers are a crucial step in facilitating the attachment of phages to their hosts [81]. Different sequences of tail fiber proteins and mutations can determine the host range variety of phages and their binding interactions [82]. Multiple tail fiber proteins help phages infect and replicate in various bacterial strains [83]. The analysis showed that this phage encodes several tail fiber proteins, resulting in the various host ranges of AEV23. As mentioned in the results, the phage encodes lysozyme R (ORF29) and Holin (ORF30), which, based on Transmembrane topology prediction results by TMHMM server, Lysozyme R was a potential outer-membrane protein and may be located on the phage tail fiber [84]. This feature can elucidate the removal of biofilm through the lysozyme/Holin system [85]. Moreover, this putative depolymerase activity can disrupt the polysaccharides of biofilms, which needs experimental validation. Understanding the specific mechanisms by which AEV23 disrupts biofilms could lead to the development of more effective phage therapies. Bacteria can eliminate and digest phage genomes through restriction enzymes and become resistant to them. However, phages that carry methyltransferase genes can evade genome digestion through restriction site methylation. Phage AEV23 has a potential methyltransferase gene (ORF76), so it can be resistant to some restriction enzymes and efficiently infect host cells. This aspect is crucial for the long-term efficacy of phage therapy, as it may reduce the likelihood of bacterial resistance developing [86]. Another immune system of bacteria is the CRISPR system, which can also digest viral genomes. The Cas4-domain exonuclease encoded by (ORF16) has 5’ to 3’ DNA activity and is involved in the CRISPR system. It has been revealed that the overexpression of Sulfolobus viral Cas4 leads to the removal of spacer acquisition and supports the anti-CRISPR role of virus-encoded Cas4 proteins, resulting in the inhibition of spacer acquisition [87]. Four ORFs encode *E. coli* genes, such as IS5 family transposase (ORF75), which is a mobile element that can act as a gene silencer for the removal of its host. Therefore, it can be concluded that the AEV23 phage, which does not encode tRNA, antibiotic resistance genes, virulence factors and integrase genes, possesses efficient genes for lysing various bacteria. Therefore, AEV23 phage can be proposed as a virulence phage against *K. pneumoniae* and supports the safety application of the AEV23 phage. The unique properties of the AEV23 phage, including its high lytic activity, offer a major therapeutic advantage by minimizing disruption to beneficial microbiota. Along with the absence of virulence factors, this identifies AEV23 as a safe and effective candidate against *K. pneumoniae*, though further studies are needed. Its ability to reduce biofilm by up to 93% is likely due to a dual enzymatic mechanism involving EPS degradation and lysozyme-holin-mediated lysis (ORF29/30), which is especially important for treating chronic CF infections where biofilms reduce antibiotic efficacy. Genomically, the AEV23 methyltransferase gene (ORF76) provides a tactical advantage by evading bacterial restriction systems, a feature rarely reported in therapeutic *Klebsiella* phages, contributing to new insights into how phages can combat multidrug-resistant pathogens and making it a valuable tool.

Clinical trials are needed to confirm the efficacy of AEV23 in CF patients, based on existing phage therapy models for *K. pneumoniae*. This study supports future research on combining AEV23 with other phages to broaden its effectiveness. Although phage resistance may occur, new phages can be identified more quickly than developing new antibiotics. While no serious side effects have been reported, safety concerns remain. Using phage cocktails from well-characterized banks that lack harmful genes and target multiple bacterial receptors may help reduce risks such as endotoxin release, gene transfer, and resistance development [88, 89]. Exploring the combination of phage therapy with existing antibiotics or other antimicrobial agents may enhance treatment effectiveness, particularly in severe infections requiring rapid intervention [90].

While this study presents the initial isolation and genomic characterization of the novel lytic phage AEV23, we acknowledge that further functional evaluations are critical to assess its full therapeutic potential. Specifically, long-term interaction studies such as time-kill curves, co-evolution assays, and receptor/capsule mutation analyses are necessary to understand resistance dynamics and host adaptation. These experiments, along with potential synergy tests with antibiotics, are currently underway in our lab and will be presented in future work. Importantly, our focus on multidrug-resistant *K. pneumoniae* strains isolates from Cystic Fibrosis patients an underrepresented yet clinically significant group adds a specific translational value to the current study. Focusing on the discovery of new phages is crucial, as they have great potential for treating patients with MDR infections through phage therapy applications, especially for Cystic Fibrosis patients.

## Conclusion

Genome analysis and characterization of phage AEV23 showed that it has high host specificity, strong lysis activity against *K. pneumoniae*. It has no human virulence factors in its genome. Therefore, it can be an appropriate candidate for the development of novel biocontrol agents to treat resistant *K. pneumoniae* infections. Further studies including host receptor identification, phage cocktail optimization, and clinical in vivo studies are needed to assess its effectiveness.

## Data Availability

The genomic sequence of AEV23 generated during this study is available at GenBank (accession number: OR810919.). All other datasets generated during and/or analyzed during the current study are available from the corresponding author upon reasonable request.
